# Efficacy and Safety of Chinese Patent Medicine Combined With Oseltamivir in Treatment of Children With Influenza: A meta-Analysis

**DOI:** 10.3389/fphar.2021.682732

**Published:** 2021-08-06

**Authors:** Nai-fan Duan, Bin Liu, Xiao-na Li, Yi-bai Xiong, Yan Zhang, Chi Zhang, Li LI, Cheng Lu, Jueni Lyu

**Affiliations:** ^1^ Institute of Basic Research in Clinical Medicine, China Academy of Chinese Medical Science, Beijing, China; ^2^ China Center for Evidence Based Traditional Chinese Medicine, Beijing, China; ^3^ Dongzhimen Hospital, Beijing University of Chinese Medicine, Beijing, China; ^4^ HKU Business School, The University of Hong Kong, Pok Fu Lam, Hong Kong

**Keywords:** influenza, children, Chinese patent medicine, oseltamivir, meta-analysis

## Abstract

**Background:** Recently, Chinese patent medicines (CPMs) have been widely used to treat children with influenza in China, with curative effects. Therefore, the efficacy and safety of such treatment require further evaluation. The present meta-analysis integrated data from several independent studies to determine overall treatment trends in children with influenza.

**Methods:** The following databases were searched for randomized controlled trials (RCTs) published from their inception to December 12, 2020: CNKI, Wanfang, SinoMed, PubMed, Cochrane library, and Embase. Two researchers independently extracted the data, assessed the methodological quality of the studies, and conducted a meta-analysis of the results using Review Manager 5.2. The results were assessed using forest plots, and publication bias was evaluated using a funnel plot.

**Results:** A total of 21 RCTs involving 2960 cases were included. Compared to oseltamivir alone, CPMs combined with oseltamivir reduced the duration of symptoms, including that of fever (mean difference [MD] = −0.64, 95% confidence interval [CI]: −0.86 to −0.41, *P <* 0.00001), cough (MD = −0.82, 95% CI: −1.02 to −0.62, *P* < 0.00001), nasal obstruction (MD = −0.88, 95% CI: −1.15 to −0.61, *P* < 0.00001), and sore throat (MD = −0.92, 95% CI: −1.26 to −0.57, *P* < 0.00001). Combined therapy also reduced the time of viral shedding (MD = −0.53, 95% CI: −0.70 to −0.36, *P* < 0.00001) and the occurrence of adverse drug reactions (ADRs) (RR=0.53, 95% CI: 0.34 to 0.83, *P* = 0.005).

**Conclusions:** CPMs combined with oseltamivir reduced the duration of symptoms, shortened the time of viral shedding, and reduced the number of ADRs. However, these results should be considered with caution because there was marked heterogeneity and publication bias in the research data. More rigorous RCTs should be designed to verify the effect of CPMs in children with influenza.

## Introduction

Influenza is an acute respiratory infectious disease caused by influenza virus. The disease is common worldwide and its main symptoms include fever, cough, nasal congestion, and sore throat (National Health Commission of the People’s Republic of China, 2018). During the influenza season, children are particularly vulnerable. According to data from the World Health Organization, the annual incidence of influenza in children ranges from 20 to 30% worldwide. In severe influenza seasons, the annual influenza infection rate in children can be as high as 50% (COMMITTEE ON INFECTIOUS DISEASES, 2018). Generally, children with influenza are treated using oseltamivir, peramivir, or zanamivir, with oseltamivir being the most commonly used (COMMITTEE ON INFECTIOUS DISEASES, 2019). Oseltamivir carboxylate, the active metabolite of oseltamivir, inhibits the neuraminidase activity of influenza virus and reduces transmission by preventing the release of the virus from infected cells. The best effect is achieved when drug treatment is initiated within 24 h of symptom onset. However, influenza virus has gradually developed resistance to oseltamivir (Govorkova et al., 2001; Kiso et al., 2004; Moscona, 2004; Moscona, 2009; Panning, 2013; Lina et al., 2018), and some patients have shown adverse drug reactions (ADRs) (Zhou et al., 2019). Therefore, oseltamivir may not remain a viable treatment for influenza in the long run, and new therapies should be developed to improve influenza treatment in children and reduce the use of antiviral drugs.

In Chinese patent medicines (CPMs), traditional Chinese medicine (TCM) based raw materials are processed into defined dosages and forms according to prescriptions. They are chemically stable; have a definite curative effect with relatively less toxicity and side effects; and can be carried and stored easily (Zhang, 2018). In general, children infected with influenza mostly manifest mild symptoms, such as fever, respiratory symptoms such as cough, sore throat, runny nose, and nasal congestion. A small proportion of children manifest gastrointestinal symptoms, such as nausea, vomiting, and diarrhea. The clinical symptoms of influenza in infants are often atypical. Neonatal influenza is relatively rare, but it can lead to pneumonia and symptoms of sepsis, such as lethargy, refusal to eat, and apnea. Children of different ages present different clinical manifestations at different stages of influenza. Many types of CPMs are used to treat influenza in children at different ages and disease stages (Cao et al., 2015). Such treatments have marked advantages and significant effects, reducing symptoms, shortening the disease course, and reducing complications (Yao et al., 2015; Rong et al., 2017; Wu 2018). Moreover, several RCTs have indicated that CPMs combined with oseltamivir have some curative effects in children with influenza (Xiong et al., 2020). For example, *Xiaoer Chiqiao Qingre* granules are generally used to treat children with symptoms such as fever, cough, nasal congestion, sore throat, and constipation. Evidence shows that *Xiaoer Chiqiao Qingre* granules can improve symptoms in children with acute upper respiratory tract infections and reduce the levels of inflammatory factors in the serum of these children; hence, their use can be promoted in clinical practice (Wang, 2019). Another study has shown that *Xiaoer Chiqiao Qingre* granules have a significant antipyretic effect in children with viral upper respiratory tract infections, quickening symptom improvement and showing high drug safety (Han et al., 2018). In another investigation, *Lianhua Qingwen* granules combined with oseltamivir have been shown to shorten the time to fever resolution, reduce the duration of symptoms. The children tolerated the combined treatment, hence, they can be used in clinical practice (Wang et al., 2020). *Yinqiao San* consists of Chinese medicines, such as *Lonicera japonica* (Thunb) and *Forsythia suspensa* (Thunb, Vahl). Studies have shown that it has inhibitory effects on various viruses. In animal experiments, it slightly protected mice from influenza A virus infection. Among the 17 chemical components separated from *Yinqiao San*, the main antiviral active components were identified as lignans and flavonoids. Specifically, the active components liquiritin and arctiin have relatively weak antiviral effects, reflecting the synergistic effect of TCM prescriptions (Shi et al., 2003). To a certain extent, the above research conclusions provide evidence for the use of CPMs in the treatment of children with influenza.

As the current clinical evidence is relatively scattered, no systematic evidence is available to support the role of CPMs in influenza treatment. The present study gathered and comprehensively evaluated data from clinical trials to increase the level of evidence and, thus, better evaluate the efficacy and safety of CPMs combined with oseltamivir in the treatment of children with influenza. In brief, we conducted a meta-analysis of the available published clinical evidence.

## Methods

### Study Registration

This systematic review was registered in the PROSPERO (registration number: CRD42020188184).

### Search Strategy

The following databases were searched from their inception to December 12, 2020: CNKI, Wanfang, SinoMed, PubMed, Cochrane library, and Embase. The following terms were searched in the abstract or title of the study (Influenza, Human OR Human Influenzas OR Influenzas, Human OR Influenza OR Influenzas OR Human Flu OR Flu, Human OR Human Influenza OR Influenza in Humans OR Influenza in Human OR Grippe) AND (Medicine, Chinese Traditional OR Traditional Chinese Medicine OR Traditional Medicine, Chinese OR Zhong Yi Xue OR Chinese Traditional Medicine OR Chinese Medicine, Traditional OR Chinese patent medicine OR Chinese Proprietary Medicine) AND (Oseltamivir OR GS 4104 OR GS4104 OR GS-4104 OR Tamiflu OR GS 4071 OR GS4071 OR GS-4071). We also manually searched for studies that met our inclusion criteria from other sources that were not included in the aforementioned databases. Two researchers (Duan N. F. and Liu B.) independently selected the eligible studies. The studies were retrieved in any language.

### Inclusion and Exclusion Criteria

This systematic review was conducted according to the Preferred Reporting Items for Systematic Review and Meta-Analysis Statement (Moher et al., 2009). The inclusion criteria were as follows: 1) RCT study design; 2) patient age >1 year and <14 years (Cao et al., 2015; Geneva, 2017); 3) treatment group treated using oral CPMs combined with oseltamivir and control group treated with oseltamivir alone; 4) main outcome of time to fever resolution; secondary outcomes of duration of cough, nasal congestion, and sore throat and time of viral shedding; and safety outcome of any ADR; 5) meeting the influenza diagnostic criteria as follows: fever accompanied by acute respiratory symptoms, such as cough, nasal congestion, or sore throat, and positive nucleic acid test results; 6) time of <48 h from symptom onset to randomization (Ma et al., 2015); 7) twice daily oral oseltamivir dosage of 30 mg in patients weighing <15 kg, 45 mg in those weighing 15–23 kg, 60 mg in those weighing 24–40 kg, and 75 mg in those weighing >41 kg; 8) treatment course of 3–7 days (Cao et al., 2015). The exclusion criteria were as follows: 1) other antiviral drugs; 2) CPM injections; 3) outcome data format that failed to meet the requirements of the statistical analysis; 4) missing data on the key outcome of time to fever resolution.

### Data Extraction and Risk of Bias Assessment

According to standard information extraction tables, two researchers (Duan N. F. and Liu B.) independently extracted the data. Throughout the process, disagreements were resolved by discussion or by involving another researcher (Lu C.). The basic information extracted from the articles included data on authors’ names, publication year, published region, type of study design, virus detection results, number of cases, age, time from symptom onset to randomization, course of treatment, randomization method, patient blinding method, researcher blinding method, inclusion and exclusion criteria, diagnostic criteria, efficacy evaluation criteria, therapeutic schedule, outcome indicators, and ADRs.

Two reviewers (Duan N. F. and Liu B.) independently assessed the risk of bias in each study using the criteria outlined in the Cochrane Handbook, (2019). Any disagreements were resolved by discussion or by involving another author (Lu C.). The risk of bias was assessed according to the following domains: 1) random sequence generation; 2) attrition bias; 3) allocation concealments; 4) blinding of participants and personnel; 5) blinding of outcome assessment; 6) incomplete outcome data; 7) selective outcome reporting; 8) other biases. Each potential source of bias was graded as high, low, or unclear, providing a quote from the study report and a justification of our judgment in the “risk of bias” table. In the table, red represents high risk, yellow represents unclear risk, and green represents low risk. We also added notes in the table when information on the risk of bias was related to unpublished data or correspondence with a trial author. When evaluating treatment effects, we considered the risk of bias in studies that contributed to the outcome.

### Data Synthesis and Analysis

Review Manager 5.2 software was used to produce the risk of bias summary and calculate a summary statistic for each outcome in the meta-analysis. Risk ratios (RRs), Mantel–Haenszel tests, and 95% confidence intervals (CI) were used to analyze dichotomous data. Mean difference (MD), inverse variance, and 95% CI were used to determine continuous variables. The *I* ^*2*^ values ranged from 0 to 100% and were categorized as follows: *I* ^*2*^ <40%, might not be important; 30% < *I* ^*2*^ <60%, moderate heterogeneity; 50% <*I* ^*2*^ <90%, substantial heterogeneity; and 75% < *I* ^*2*^ <100%, considerable heterogeneity (Higgins and Green, 2011). A fixed-effect model was used to pool the estimates. Potential sources of heterogeneity were identified using subgroup and sensitivity analyses. A random effects model was used to aggregate the results and, thus, minimize potential clinical heterogeneity. Differences were considered statistically significant at *p*-values of <0.05. We conducted a subgroup analysis of each CPM (Chinese patent medicine). The results are presented as forest plots. A funnel chart was used to analyze publication bias.

## Results

### Search Results and Study Characteristics

**FIGURE 1:**
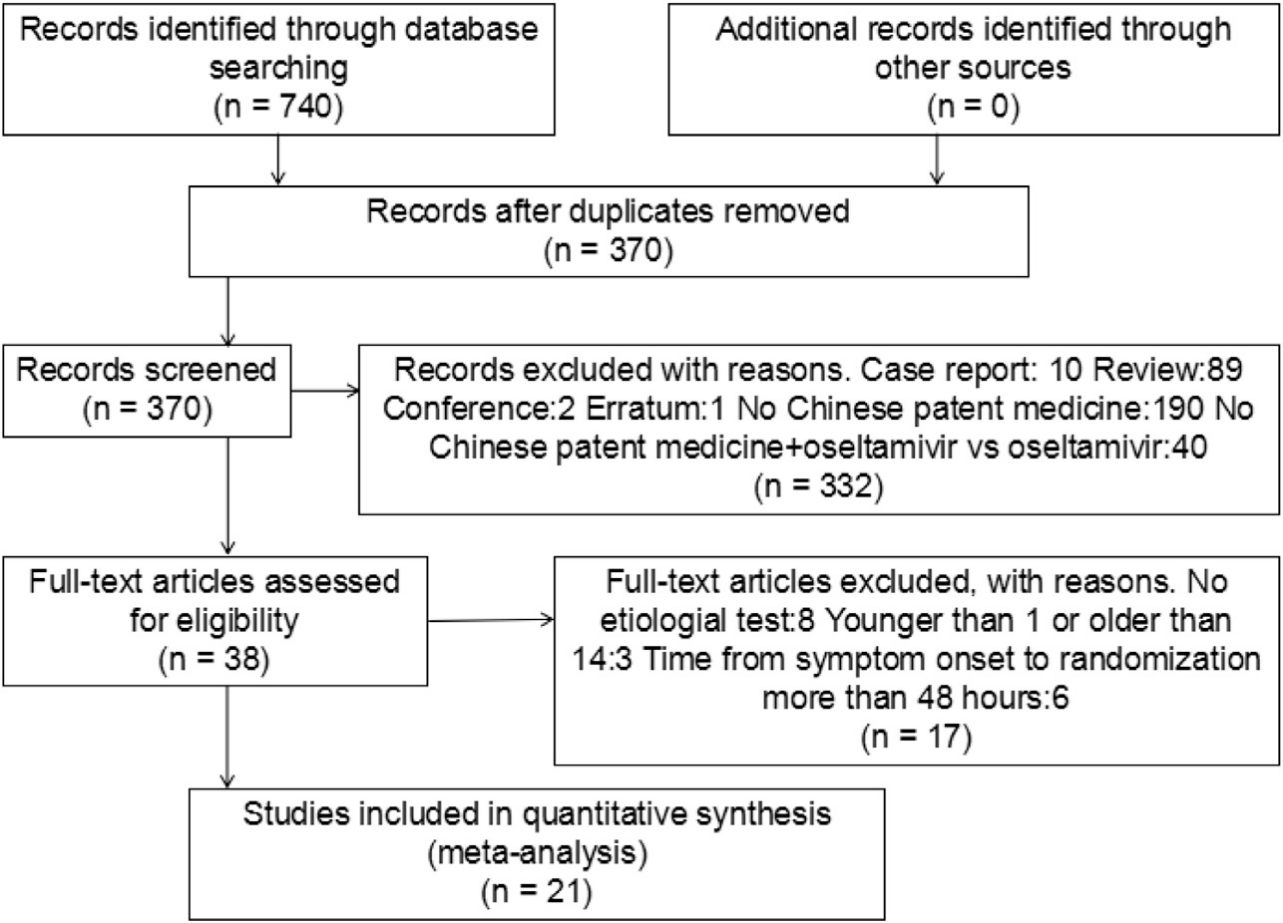
Flow chart of including and excluding studies.

A total of 740 studies were identified using the search strategy, including 160 from CNKI, 284 from Wanfang, 194 from SinoMed, 14 from PubMed, 4 from the Cochrane library, and 84 from embase. Of these 370 duplicate studies were excluded, and 332 more studies were excluded after abstract review. Of the remaining 38 studies, 17 were excluded after full text review. Ultimately, 21 RCTs involving 2,960 cases were included (Song and Zhang, 2018; Zhang, B. Y. et al., 2018; Zhang, Y. H. et al., 2018; Su, 2019; Yin, 2019; Zhou, 2019; Zhao et al., 2019; Long, 2020; Liu, 2020; Qian, 2020; Wu, 2020; Chen et al., 2019; Li, 2019; Yan, 2020; Zhu et al., 2019; Liu et al., 2020; Fang, 2018; Liu, 2018; Gao and Wang, 2020; Kuang and Wang, 2020; Du et al., 2020). The flowchart of the screening process is presented in Figure 1. Two researchers independently extracted the data from the literature. There were 1,485 and 1,475 cases in the treatment and control groups, respectively. All 21 RCTs were published in China and included seven kinds of CPMs: *Xiaoer Chiqiao Qingre* granules, *Kanggan* granules, *Lianhua Qingwen* granules, *Xiaoer Resuqing* granules (oral liquid), *Xiaoer Shuanghuanglian* mixture, *Siji Kangbingdu* mixture, and *Xiaoer Niuhuang Qingxin* powder. The data were published between 2018 and 2020. All cases tested positive for influenza virus. The treatment course was 3–7 days. Nine studies reported ADRs (Zhang, B. Y. et al., 2018; Yin, 2019; Zhou, 2019; Zhao et al., 2019; Long, 2020; Liu, 2020; Qian, 2020; Wu, 2020; Gao and Wang, 2020). The baseline characteristics were consistent across the studies. The detailed characteristics of the studies are presented in Table 1.

**TABLE 1:** Characteristics of included studies.

Study year region	Type	Types of Chinese patent medicine	Influenza virus	Cases (Treatment group/Control group)	Age (Treatment group/Control group)	Time from symptom onset to randomization (h)	Dosage		Course of treatment	Outcomes	Adverse reactions	
							CPMs	Oseltamivir			Treatment group	Control group
Song (2018) China	RCT	Xiaoer Chiqiao Qingre Granules	+	68/60	1∼14(6.93 ± 4.26)/1∼14(5.13 ± 4.82)	2∼48(15.31 ± 9.54)/2∼ 48(13.22 ± 7.21)	1∼3 years old, 2∼3 g/time; 4∼6 years old, 3∼4 g/time; 7∼9 years old, 4∼5 g/time; >10 years old, 6 g/time; 3 times/d	<15 kg 30 mg/time, 15∼23 kg 45 mg/time, 24∼40 kg 60 mg/time, >41 kg 75 mg/time, 2 times/d	5	A13	NA	NA
Zhang (2018) China	RCT	Xiaoer Chiqiao Qingre Granules	+	46/46	1∼12(7 ± 2.6)/1∼12(6.4 ± 2.5)	(18.4 ± 7)/(19 ± 7.2)	1∼3 years old, 2∼3 g/time; 4∼6 years old, 3∼4g/time; 7∼9 years old, 4∼5 g/time; >10 years old, 6 g/time; 3 times/d	<15 kg 30 mg/time, 15∼23 kg 45 mg/time, 24∼40 kg 60 mg/time, >41 kg 75 mg/time, 2 times/d	5	A1235	3 cases of nausea,vomiting and diarrhea	3 cases of nausea,vomiting and diarrhea 2 cases of diarrhea
Zhangy (2018) China	RCT	Xiaoer Chiqiao Qingre Granules	+	57/57	1∼12(3.5 ± 0.53)/1∼12(3.5 ± 0.53)	(36.96 ± 10.08)/(36.96 ± 10.08)	1∼3 years old, 2 g/time; 4∼6 years old, 4 g/time; 7∼9 years old, 5 g/time; >10 years old, 6 g/time; 3 times/d	<15 kg 30 mg/time, 15∼23 kg 45 mg/time, 24∼40 kg 60 mg/time, >41 kg 75 mg/time, 2 times/d	5	A	NA	NA
Su (2011) China	RCT	Xiaoer Chiqiao Qingre Granules	+	53/53	1∼12(6.7 ± 3.2)/1∼13(6.9 ± 3.1)	5∼35(19.5 ± 3.8)/7∼36(19.8 ± 3.6)	<3 years old 2 g/time, 3∼6 years old 3 g/time, 7∼10 years old 4 g/time, >11 years old 6 g/time, 3 times/d	<15 kg 30 mg/time, 15∼2 3kg 45 mg/time, 24∼40 kg 60 mg/time, >41 kg 75 mg/time, 2 times/d	5	A12	NA	NA
Yin (2011) China	RCT	Xiaoer Chiqiao Qingre Granules	+	30/30	1∼10(5.2 ± 1.3)/1∼9(4.9 ± 1.6)	6∼35/6∼32	1∼3 years old, 2∼3 g/time, 4∼6 years old, 3∼4 g/time, 7∼9 years old, 4∼5 g/time, >10 years old, 6 g/time, 3 times/d	<15 kg 30 mg/time, 15∼23 kg 45 mg/time, 24∼40 kg 60 mg/time, >41 kg 75 mg/time, 2 times/d	5	A135	1 case of nausea and vomiting. 1 case of abdominal pain	2 cases of nausea and vomiting. 1 case of diarrhea
Zhou (2019) China	RCT	Xiaoer Chiqiao Qingre Granules	+	118/118	1∼12(6.5 ± 0.7)/1∼12 (6.8 ± 0.5)	3∼47(22.8 ± 3.5)/4∼48(22.5 ± 3.7)	<3 years old, 2∼3 g/time; 3∼6 years old, 3∼4 g/time; 6∼10 years old, 4∼5 g/time; >10 years old, 6 g/time, 3 times/d	<15 kg 30 mg/time, 15∼23 kg 45 mg/time, 24∼40 kg 60 mg/time, >41 kg 75 mg/time, 2 times/d	5	A45	2 cases of nausea and vomiting. 2 cases of abdominal pain	4 cases of nausea and vomiting. 4 cases of abdominal pain. 2 cases of diarrhea
Zhau (2019) China	RCT	Xiaoer Chiqiao Qingre Granules	+	75/75	1∼14(7.2 ± 3.5)/1∼13(7.8 ± 4.1)	7∼37(19.67 ± 9.56)/9∼36(19.28 ± 8.97)	1∼3 years old, 2∼3 g/time, 4∼6 years old, 3∼4 g/time, 7∼9 years old, 4∼5 g/time, >10 years old, 6 g/time, 3 times/d	<15 kg 30 mg/time, 15∼23 kg 45 mg/time, 24∼40 kg 60 mg/time, >41 kg 75 mg/time, 2 times/d	5	A125	3 cases of nausea and vomiting. 1 case of abdominal pain. 2 cases of diarrhea	4 cases of nausea and vomiting. 3 cases of abdominal pain. 1 case of diarrhea
Long (2020) China	RCT	Xiaoer Chiqiao Qingre Granules	+	92/92	(6.5 ± 0.7)/(6.4 ± 0.9)	(19.06 ± 0.61)/(19.16 ± 0.51)	1∼3 years old, 2∼3 g/time, 4∼6 years old, 3∼4 g/time, 7∼9 years old, 4∼5 g/time, >10 years old, 6 g/time, 3 times/d	<15 kg 30 mg/time, 15∼23 kg 45 mg/time, 24∼40 kg 60 mg/time, >41 kg 75 mg/time, 2 times/d	5	A1235	1 case of nausea. 1case of vomiting. 1 case of diarrhea	2 cases of nausea. 1 case of vomiting. 1 case of diarrhea. 1 case of abdominal pain
Liu (2020) China	RCT	Xiaoer Chiqiao Qingre Granules	+	69/69	1∼13(7.4 ± 1.9)/1∼11(7.3 ± 1.8)	7∼42(18.6 ± 4.4)/6∼39(18.3 ± 4.2)	1∼3 years old, 2∼3 g/time, 4∼6 years old, 3∼4 g/time, 7∼9 years old, 4∼5 g/time, >10 years old, 6 g/time, 3 times/d	<15 kg 30 mg/time, 15∼23 kg 45 mg/time, 24∼40 kg 60 mg/time, >41 kg 75 mg/time, 2 times/d	5	A1235	2 cases of nausea. 1 case of diarrhea	2 cases of nausea. 2 cases of diarrhea
Qian (2020) China	RCT	Xiaoer Chiqiao Qingre Granules	+	50/50	2∼12(7.2 ± 1.2)/1∼12(7.4 ± 1.2)	<48/<48	1∼3 years old, 2∼3 g/time, 4∼6 years old, 3∼4 g/time, 7∼9 years old, 4∼5 g/time, >10 years old, 6 g/time, 3 times/d	<15 kg 30 mg/time, 15∼23 kg 45 mg/time, 24∼40 kg 60 mg/time, >41 kg 75 mg/time, 2 times/d	5	A125	1 case of nausea and vomiting. 1 case of abdominal pain	4 cases of nausea and vomiting. 3 cases of abdominal pain. 1 case of diarrhea
Wu (2020) China	RCT	Xiaoer Chiqiao Qingre Granules	+	33/32	1∼13(6.7 ± 2.1)/1∼12(6.6 ± 2.1)	7∼35(21.2 ± 4.1)/7∼35(21.2 ± 4.1)	1∼3 years old, 2∼3 g/time, 4∼6 years old, 3∼4 g/time, 7∼9 years old, 4∼5 g/time, >10 years old, 6 g/time, 3 times/d	<15 kg 30 mg/time, 15∼23 kg 45 mg/time, 24∼40 kg 60 mg/time, >41 kg 75 mg/time, 2 times/d	5	A235	1 case of nausea. 1 case of diarrhea	3 cases of nausea. 5 cases of diarrhea
Chen (2019) China	RCT	Kanggan Granules	+	31/31	1∼14/1∼14	3∼45(22.1 ± 6.0)/5∼48(22.8 ± 6.3)	1∼5 years old, 2.5 g/time, 6∼9 years old, 5 g/time, 10∼14 years old, 7.5 g/time,3 times/d	<15 kg 30 mg/time, 15∼23 kg 45 mg/time, 24∼40 kg 60 mg/time, >41 kg 75 mg/time, 2 times/d	5	A	NA	NA
Li (2019) China	RCT	Kanggan Granules	+	193/193	2∼6/2∼6	<48/<48	1∼5 years old, 2.5 g/time, 6∼9 years old, 5 g/time, 3 times/d	<15 kg 30 mg/time, 15∼23 kg 45 mg/time, 24∼40 kg 60 mg/time, >41 kg 75 mg/time, 2 times/d	5	A	NA	NA
Yan (2020) China	RCT	Kanggan Granules	+	45/45	1∼10(5.15 ± 2.19)/1∼11 (5.19 ± 2.1)	3∼43(23.52 ± 4.81)/3∼45(23.82 ± 4.83)	1∼3 years old, 2.5 g/time, 4∼7 years old, 5 g/time, 8∼11 years old, 7.5 g/time,3 times/d	<15 kg 30 mg/time, 15∼23 kg 45 mg/time, 24∼40 kg 60 mg/time, >41 kg 75 mg/time, 2 times/d	7	23	NA	NA
Zhu (2019) China	RCT	Lianhua Qingwen Granules	+	110/110	3∼13(9.10 ± 2.06)/3∼14(9.03 ± 2.12)	4∼48(22.08 ± 9.52)/3∼46(21.35 ± 9.64)	≤23 kg 3 g/time, >23 kg 6 g/time, 3 times/d	<15 kg 30 mg/time, 15∼23 kg 45 mg/time, 24∼40 kg 60 mg/time, >41 kg 75 mg/time, 2 times/d	3	A134	NA	NA
Liu 2020 China	RCT	Lianhua Qingwen Granules	+	34/34	(4.78 ± 1.39)/(4.76 ± 1.33)	(19.64 ± 4.72)/(18.65 ± 4.32)	1∼6 years old, 3 g/time,6∼8 years old, 6 g/time, 3 times/d	<15 kg 30 mg/time, 15∼23 kg 45 mg/time, 24∼40 kg 60 mg/time, 2 times/d	7	A13	NA	NA
Fang (2018) China	RCT	Xiaoer Resuqing Granules	+	66/65	2∼12 (5.81 ± 2.73)/2∼12 (5.63 ± 2.95)	1∼46(20.45 ± 12.77)/1∼46 (18.78 ± 10.52)	1∼3 years old, 1∼2 g/time, 4∼6 years old, 2∼3 g/time, 7∼12 years old, 3∼4 g/time, 3∼4 times/d	<15 kg 30 mg/time, 15∼23 kg 45 mg/time, 24∼40 kg 60 mg/time, >41 kg 75 mg/time, 2 times/d	3	A	NA	NA
Liu (2018) China	RCT	Xiaoer Resuqing oral liquid	+	85/85	2∼11(6.54 ± 2.22)/1∼12(6.57 ± 2.19)	4∼37(19.26 ± 3.49)/6∼35(19.31 ± 3.54)	1∼3 years old, 1∼2 g/time, 4∼7 years old, 2∼3 g/time, 8∼12 years old, 3∼4 g/time, 3 times/d	<15 kg 30 mg/time, 15∼23 kg 45 mg/time, 24∼40 kg 60 mg/time, >41 kg 75 mg/time, 2 times/d	7	1234	NA	NA
Gao (2020) China	RCT	Xiaoer Shuanghuanglian Mixture	+	45/45	3∼10(5.1 ± 2.4)/2∼11(5.3 ± 2.0)	12∼48(28.8 ± 2.4) 7.2∼48(31.2 ± 4.8)	1∼3 years old, 10 ml/time, >3 years old, 20 ml/time, 3 times/d	<15 kg 30 mg/time, 15∼23 kg 45 mg/time, 24∼40 kg 60 mg/time, >41 kg 75 mg/time, 2 times/d	7	A15	1 case of abdominal distension 1 case of skin pruritus	1 case of diarrhea
Kuang (2020) China	RCT	Siji Kangbingdu Mixture	+	110/110	1∼12(6.50 ± 3.41)/1∼12(6.49 ± 3.39)	2∼48(25.44 ± 8.20)/3∼48(25.47 ± 8. 21)	<2 years old, 3∼5 ml/time, 2∼5 years old, 5 ml/time, 5∼7 years old, 5∼10 ml/time, >7 years old, 10∼20 ml/time, 3 times/d	<15 kg 30 mg/time, 15∼23 kg 45 mg/time, 24∼40 kg 60 mg/time, >41 kg 75 mg/time, 2 times/d	5	A12	NA	NA
Du (2020) China	RCT	Xiaoer Niuhuang Qingxin Powder	+	75/75	2∼6(3.8 ± 2.46)/2∼6(3.69 ± 2.17)	8∼45(18.05 ± 6.17)/6∼48 (18.33 ± 6.29)	<1 years old, 0.15 g/time, 1∼3 years old, 0.3 g/time, 3∼8 years old, 0.45 g/time, 2 times/d	<15 kg 30 mg/time, 15∼23 kg 45 mg/time, 24∼40 kg 60 mg/time, >41 kg 75 mg/time, 2 times/d	5	A123	NA	NA

Notes:

The “+” means positive for the influenza virus.

The outcomes are time of defervescence, the easing time of cough, the easing time of nasal obstruction, the easing time of sore throat, the time of viral shedding and adverse reactions, that are denoted by “A” “1” “2” “3” “4” and “5” respectively.

### Methodological Quality Assessment

**FIGURE 2:**
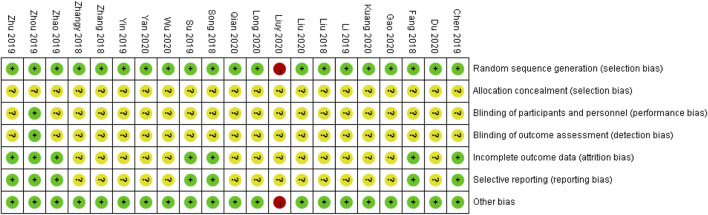
Assessment of risk of bias in the 21 trials.

Assessment of the risk of bias in the 21 trials (Figure 2 **)** showed that all studies implemented randomized grouping, 12 trials used random number tables, while the remaining nine trials did not report a specific random allocation method. One study (Zhou, 2019) used a blinding method, but it was unclear whether single or double blinding was implemented. The remaining studies did not provide blinding information. Regarding allocation concealment, 12 studies used a random number table method (Zhang, B. Y. et al., 2018; Su, 2019; Zhao et al., 2019; Long, 2020; Chen et al., 2019; Zhu et al., 2019; Liu et al., 2020; Fang, 2018; Liu, 2018; Gao and Wang, 2020; Kuang and Wang, 2020; Du et al., 2020), but it was unclear whether the randomly assigned researchers and allocation concealment researchers were third-party personnel. Therefore, the studies were rated as having an unclear risk. The remaining studies were also rated as having an unclear risk. With regard to incomplete outcome data and selective reporting, seven studies reported the results according to preset outcome indicators and were, thus, rated as having a low risk (Fang, 2018; Song and Zhang, 2018; Chen et al., 2019; Su, 2019; Zhao et al., 2019; Zhou, 2019; Zhu et al., 2019). The remaining studies failed to clarify whether the outcome indicators were established in advance and were, therefore, rated as having an unclear risk. With regard to other biases, one study was suspected of reporting plagiarized data and, therefore, was rated as having a high risk. The remaining studies showed no obvious other biases and, thus, were rated as having a low risk.

### Primary Outcome

#### Time to Fever Resolution

**FIGURE 3:**
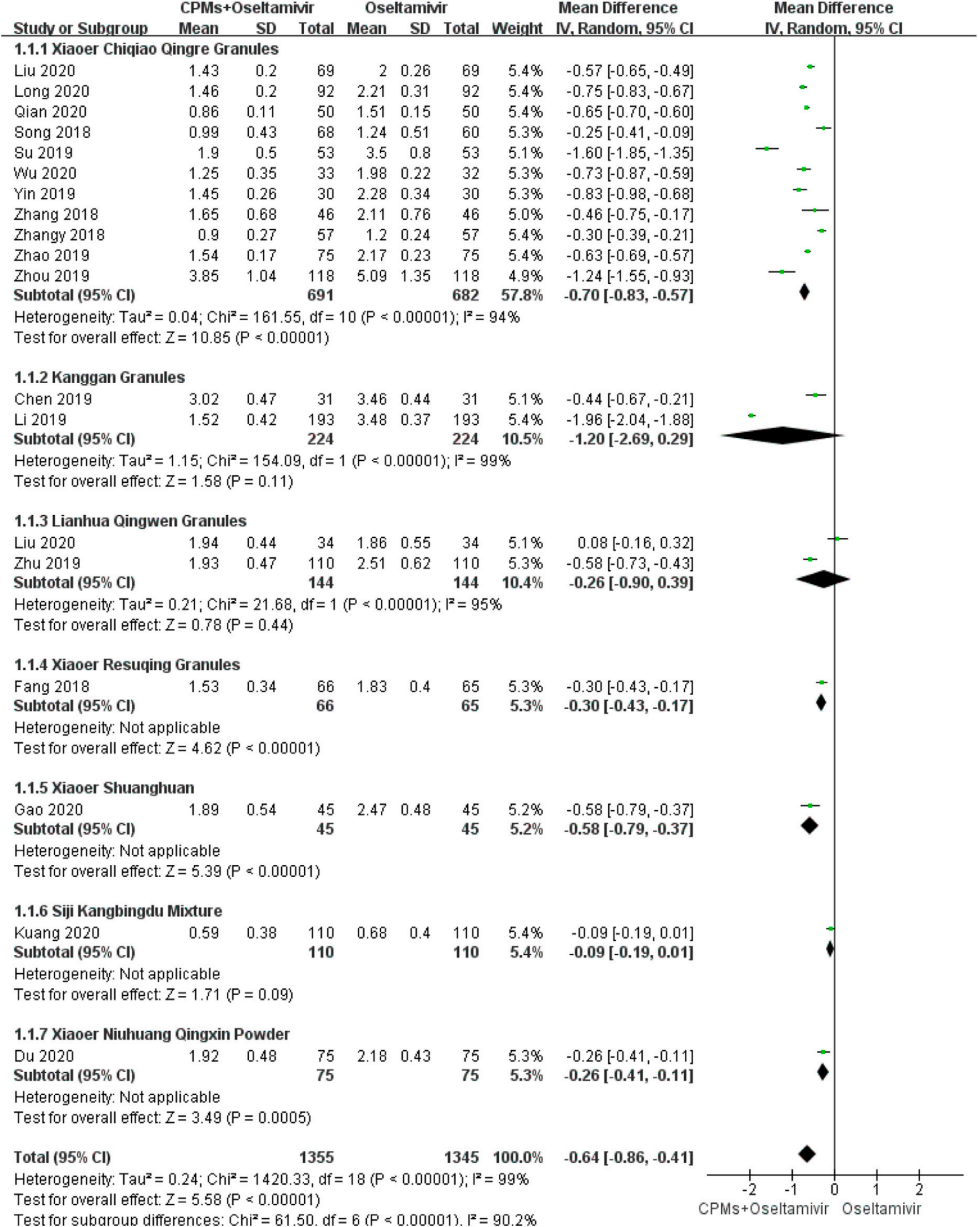
Forest plot of time to fever resolution.

Nineteen studies involving 2,700 cases reported the time to fever resolution (Song and Zhang, 2018; Zhang, B. Y. et al., 2018; Zhang, Y. H. et al., 2018; Su, 2019; Yin, 2019; Zhou, 2019; Zhao et al., 2019; Long, 2020; Liu, 2020; Qian, 2020; Wu, 2020; Chen et al., 2019; Li, 2019; Zhu et al., 2019; Liu et al., 2020; Fang, 2018; Gao and Wang, 2020; Kuang and Wang, 2020; Du et al., 2020). The forest plot showed significant differences between the treatment and control groups (MD = −0.64, 95% CI: 0.86 to −0.41, *p* < 0.00001; Figure 3). The heterogeneity was high (*I* ^*2*^ = 99%, *p* < 0.00001). A random effects model was used to analyze the data.

### Secondary Outcomes

#### Duration of Cough

**FIGURE 4:**
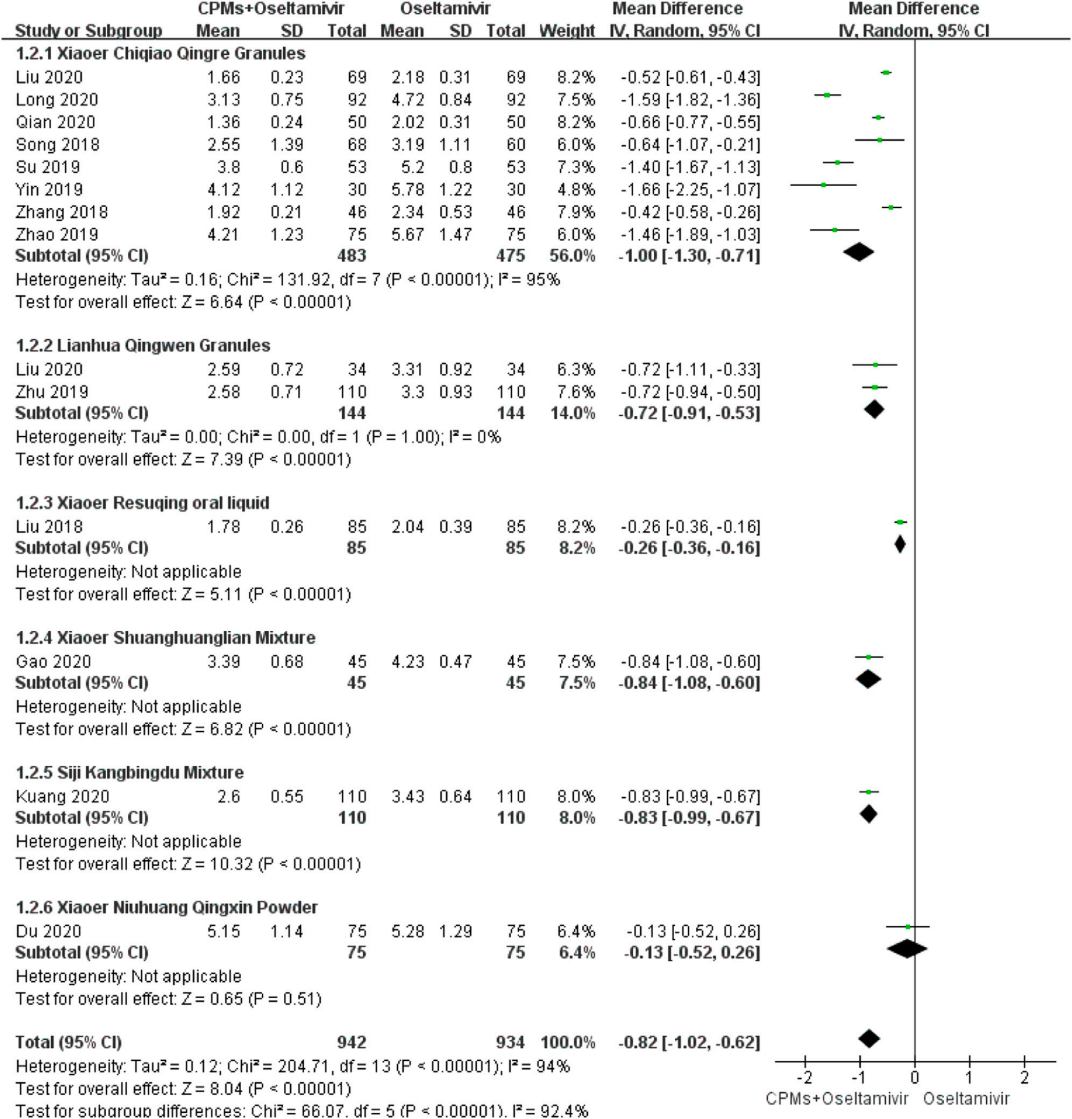
Forest plot of duration of cough.

Fourteen studies involving 1876 cases reported the duration of cough (Song and Zhang, 2018; Zhang, B. Y. et al., 2018; Su, 2019; Yin, 2019; Zhao et al., 2019; Long, 2020; Liu, 2020; Qian, 2020; Zhu et al., 2019; Liu et al., 2020; Liu, 2018; Gao and Wang, 2020; Kuang and Wang, 2020; Du et al., 2020). The forest plot (Figure 4) showed significant differences between the treatment and control groups (MD = −0.82, 95% CI: 1.02 to −0.62, *p* < 0.00001), and the heterogeneity was high (*p* < 0.00001, *I* ^*2*^ = 94%). The random effects model was used to analyze the data.

#### Duration of Nasal Congestion

**FIGURE 5:**
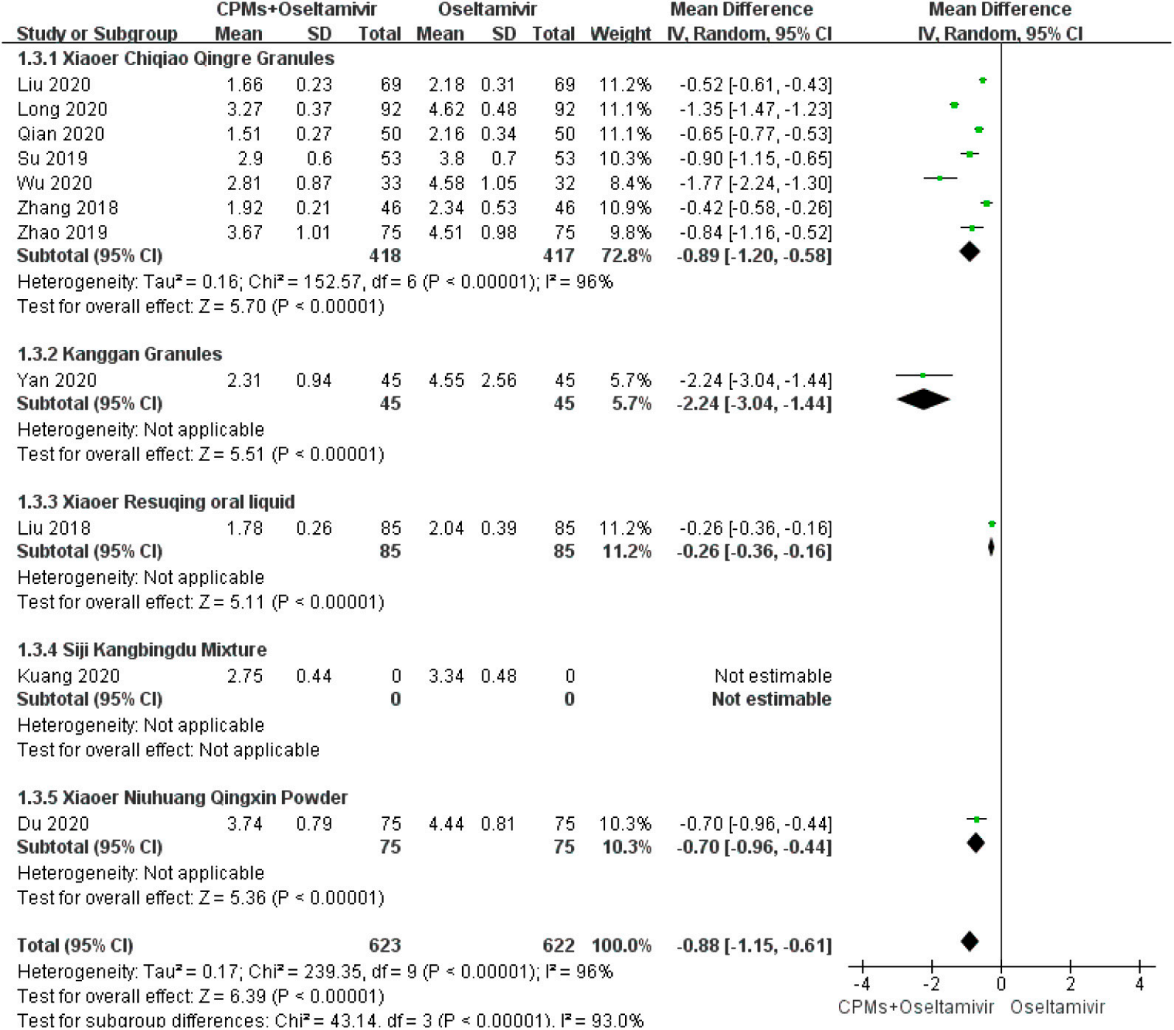
Forest plot of duration of nasal congestion.

Eleven studies involving 1,245 cases reported the duration of nasal congestion (Zhang, B. Y. et al., 2018; Su, 2019; Zhao et al., 2019; Long, 2020; Liu, 2020; Qian, 2020; Wu, 2020; Yan, 2020; Liu, 2018; Kuang and Wang, 2020; Du et al., 2020). The forest plot (Figure 5) showed significant differences between the treatment and control groups (MD = −0.88, 95% CI: 1.15 to −0.61, *p* < 0.00001), and the heterogeneity was high (*I* ^*2*^ = 96%, *p* < 0.00001). The random effects model was used to analyze the data.

#### Duration of Sore Throat

**FIGURE 6:**
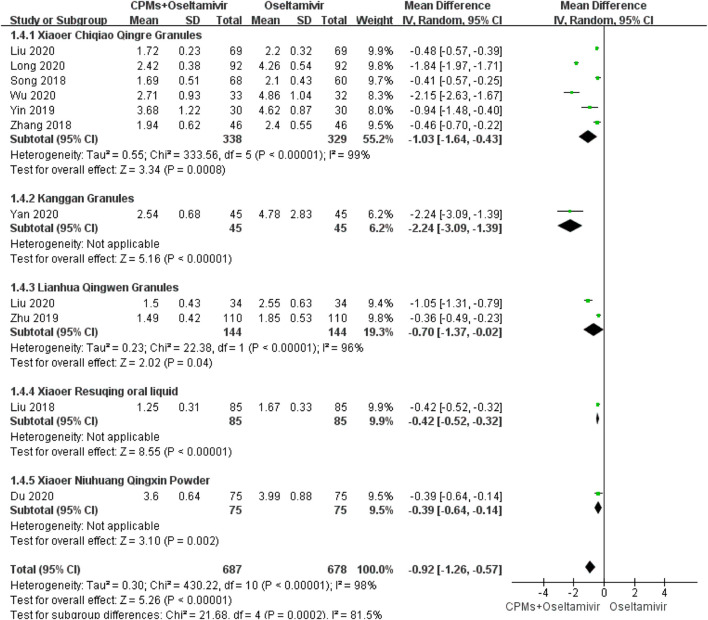
Forest plot of duration of sore throat.

Eleven studies involving 1,365 cases reported the duration of sore throat (Song and Zhang, 2018; Zhang, B. Y. et al., 2018; Yin, 2019; Long, 2020; Liu, 2020; Wu, 2020; Yan, 2020; Zhu et al., 2019; Liu, 2020; Liu, 2018; Du et al., 2020). The forest plot (Figure 6) showed significant differences between the treatment and control groups (MD = −0.92, 95% CI: 1.26 to −0.57, *p* < 0.00001), and the heterogeneity was high (*I* ^*2*^ = 98%, *p* < 0.00001). The random effects model was used to analyze the data.

#### Time of Viral Shedding

**FIGURE 7:**
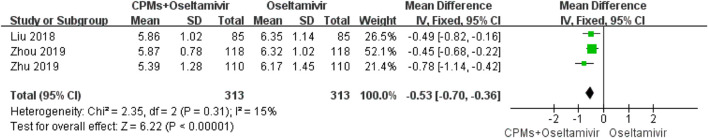
Forest plot of time of viral shedding.

Three studies involving 626 cases reported the time of viral shedding (Liu, 2018; Zhou, 2019; Zhu et al., 2019). The forest plot (Figure 7) showed significant differences between the treatment and control groups (MD = −0.53, 95% CI: 0.70 to −0.36, *p* < 0.00001). The heterogeneity was low (*I* ^*2*^ = 15%, *p* = 0.31); therefore, we used the fixed effects model to analyze the data.

### Safety Analysis: ADRs

**FIGURE 8:**
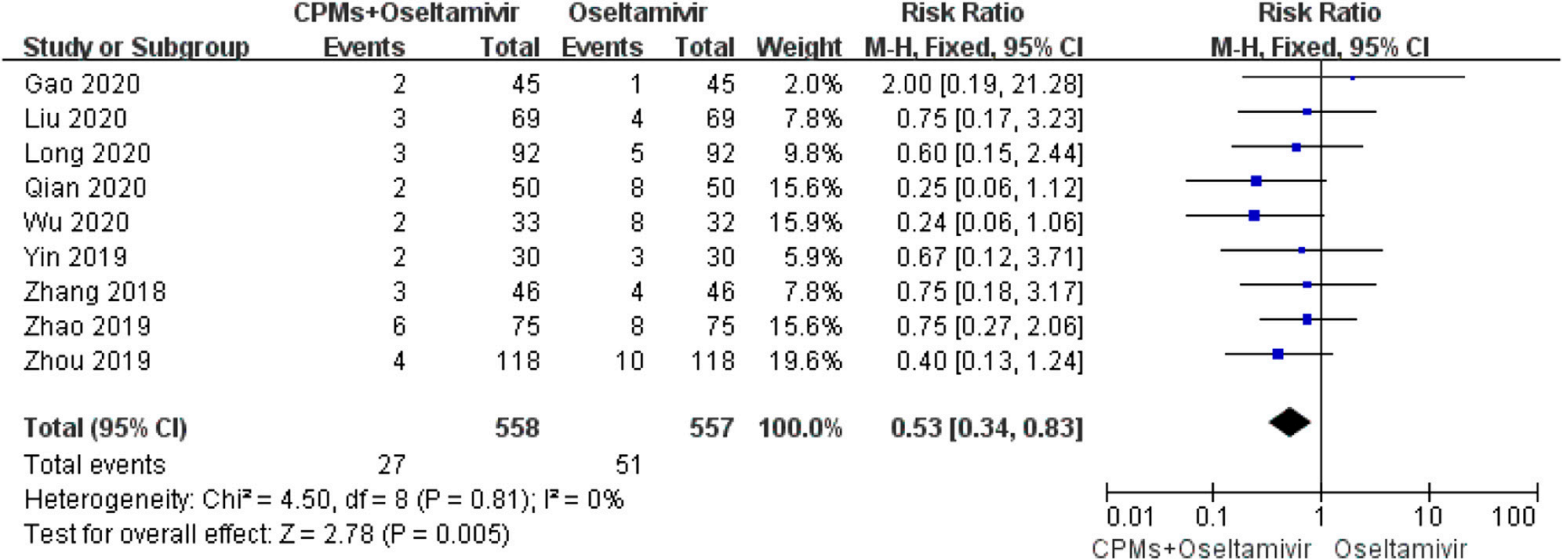
Forest plot of ADRs.

Nine studies involving 1,115 cases reported ADRs (Zhang, B. Y. et al., 2018; Yin, 2019; Zhou, 2019; Zhao et al., 2019; Long, 2020; Liu, 2020; Qian, 2020; Wu, 2020; Gao and Wang, 2020). The forest plot (Figure 8) showed significant differences between the treatment and control groups (RR = 0.53, 95% CI: 0.34 to 0.83, *p* = 0.005). There was no heterogeneity (*I* ^*2*^ = 0%, *p* = 0.81); therefore, we used the fixed effects model to analyze the data.

### Subgroup Analysis

We conducted a subgroup analysis of each CPM. There were subgroup differences in terms of time to fever resolution (90.2%), duration of cough (92.4%), nasal congestion (93%), and sore throat (81.5%). Regarding time to fever resolution, 11 studies were included in the *Xiaoer Chiqiao Qingre* granules subgroup, with 94% heterogeneity within the subgroup, two studies were included in the *Kanggan* granules subgroup, with 99% heterogeneity within the subgroup, and two were included in the *Lianhua Qingwen* granules subgroup, with 99% heterogeneity within the subgroup. Regarding duration of cough, eight studies were included in the *Xiaoer Chiqiao Qingre* granules subgroup, with 95% heterogeneity within the subgroup, and two studies were included in the *Lianhua Qingwen* granules subgroup, with 0% heterogeneity within the subgroup. Regarding duration of nasal congestion, seven studies were included in the *Xiaoer Chiqiao Qingre* granules subgroup, with 96% heterogeneity within the subgroup. Regarding duration of sore throat, six studies were included in the *Xiaoer Chiqiao Qingre* granules subgroup, with 99% heterogeneity within the subgroup. The remaining subgroups had only one study each, hence, within-group heterogeneity could not be analyzed.

### Sensitivity Analysis

Sensitivity analysis was performed to investigate potential sources of heterogeneity by observing changes in data after deletion of individual studies one by one. With regard to fever, cough, nasal congestion, and sore throat, the heterogeneity remained >90% after removal of individual studies one by one (details are shown in Supplementary Table 2). The heterogeneity results did not change significantly.

### Evaluation of Publication Bias

**FIGURE 9:**
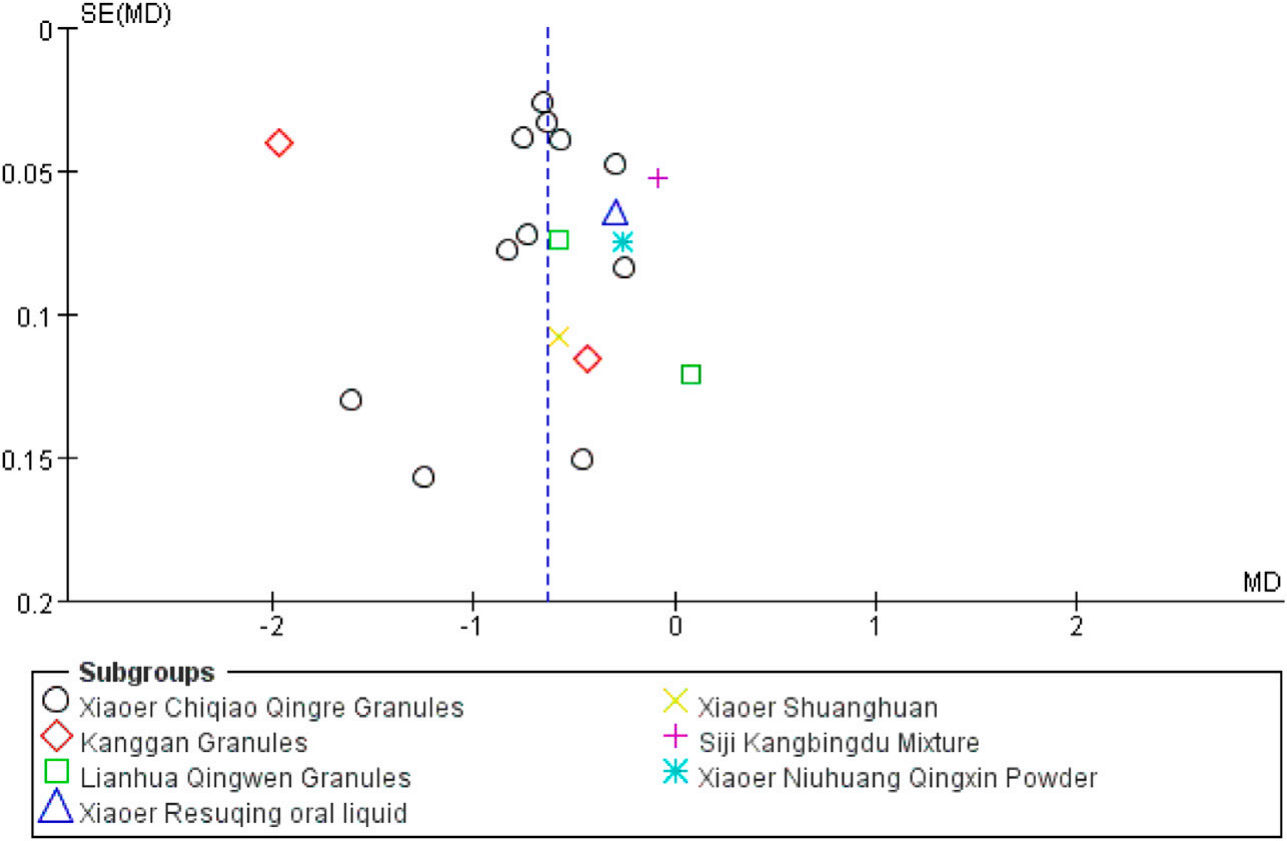
Funnel plot of time to fever resolution.

Time to fever resolution was used as the outcome; 19 studies were included (Song and Zhang, 2018; Zhang, B. Y. et al., 2018; Zhang, Y. H. et al., 2018; Su, 2019; Yin, 2019; Zhou, 2019; Zhao et al., 2019; Long, 2020; Liu, 2020; Qian, 2020; Wu, 2020; Chen et al., 2019; Li, 2019; Zhou, 2019; Liu, 2020; Fang, 2018; Gao and Wang, 2020; Kuang and Wang, 2020; Du et al., 2020), and the effect size MD was used as the horizontal axis to create a funnel chart (Figure 9). The graph was not completely symmetrical, indicating the possibility of publication bias.

## Discussion

In the present meta-analysis, the MD values for fever, cough, nasal congestion, sore throat, and viral shedding were >0.5, as shown in the forest plots, indicating that the combination of drugs shortened the course of influenza by 0.5 days. The above data reflects the trend of time reduction. In terms of ADRs, the RR was 0.53, indicating that the combination of drugs can reduce ADRs by half (Dobson et al., 2015). These results showed that, compared with oseltamivir alone, CPMs combined with oseltamivir shortened the time to fever resolution, shortened the time of symptom relief, shortened the time of viral shedding, and reduced the occurrence of ADRs, indicating that CPMs play an active role in the treatment of influenza in children. But due to the uneven quality of the current research, it will affect the reliability of the results. In order to get more accurate recommendations, we need more clinical data in the future.

However, the four outcomes of fever, cough, nasal congestion, and sore throat showed high heterogeneity. Different CPMs have different drug compositions and treatment directions, which may lead to high clinical heterogeneity (Hu et al., 2010). Therefore, we take each CPM as a subgroup and performed subgroup analysis to observe the level of heterogeneity. Differences were high among the subgroups, and subgroup analysis did not reduce the heterogeneity, possibly because there was uneven distribution among the subgroups in terms of the number of studies and cases. Except for the *Xiaoer Chiqiao Qingre* granules, *Kanggan* granules, and *Lianhua Qingwen* granules subgroups, all subgroups only included one study each; therefore, further comparative studies are needed to further evaluate the sources of heterogeneity. In the *Xiaoer Chiqiao Qingre* granules subgroup, which included the largest number of studies and cases, the heterogeneity was high. Taking time to fever resolution as an example, there were 11 studies in the *Xiaoer Chiqiao Qingre* granules subgroup, and the heterogeneity within the group was high (*p* < 0.00001, *I* ^*2*^ = 94%), possibly because the statistical unit of the outcome was time; hence, it was possible that the statistician could not accurately measure time data or that the children could not accurately express their own feelings, misleading the statistician’s judgment. Some studies did not clearly indicate whether the child was hospitalized. Moreover, when children’s data are reported by the family, the error is greater, resulting in inaccurate data (Kang et al., 2000).

We attempted to identify another source of heterogeneity. By contrast detailed characteristics of included studies, it was found that the reason for the large heterogeneity maybe due to the difference in age range and the fluctuation range of the time from onset of symptoms to randomization (Wang et al., 2009; Wang et al., 2008). The age range in the present study was 1–14 years, and the time from symptom onset to randomization was <48 h. Children of different ages have different immunity, and the time of appearance and disappearance of symptoms after infection also differs. Such a large age and time span may lead to greater data differences and increased heterogeneity. Therefore, we analyzed subgroups stratified according to age. However, in the included studies, the average age of the patients was concentrated in the 5–10 years age range, and the standard deviation values of age were uneven; hence, accurate grouping was not possible (details are shown in Supplementary Figure 10). We also planned to use time from symptom onset to randomization in subgroup analysis, but the average time was concentrated around 24 h, and the standard deviation values were uneven in some cases; hence, accurate grouping could not be performed (details are shown in Supplementary Figure 11). For both sets of data, age and time from symptom onset to randomization, the values recorded in the studies were averages; hence, we could not eldetermine the number of cases in each age subgroup and the specific time from symptom onset to randomization. More detailed information could not be obtained from the original text of each study, and no reply was received after sending the email. Both the above factors may have led to greater heterogeneity, the source of which could not be clarified. Later, we conducted sensitivity analysis and found that the heterogeneity results lacked significant changes, indicating that the included studies were relatively stable, but also that no source of heterogeneity was found. Finally, the four outcomes were combined using the random effects model. Finally, the four outcomes are combined using a random effects model. The main reason is that the RCTs of each study are not rigorous in the details of certain links. Future RCTs of children should be divided into age groups as much as possible. Every RCT should be registered. The test process and details have been carefully described to make the research more accurate and rigorous (Li et al., 2007; Liu and Xia, 2007).

To assess methodological quality, we tried to contact the corresponding authors of each study by phone or email to inquire about the details of the trials, but we did not received responses. Therefore, it was unclear whether strict operating procedures, such as allocation concealment and blinding methods, were used during the tests. The methodological quality of the included studies was poor; therefore, the reliability of the results is questionable and it is unclear whether the studies were actual RCTs (Tang et al., 1999; Moher et al., 2009).

A comprehensive analysis of the funnel chart results revealed publication bias. There were gaps in the upper left and lower right corners of the graph, indicating that some negative results were not published. In a further search for unpublished studies, we found no unpublished negative results in previous studies, possibly because there is a lack of awareness of registration in clinical research or because there are limited studies on this subject, making it impossible to obtain comprehensive evidence and, thus, leading to publication bias.

The composition of CPMs is diverse (details are shown in Supplementary Table 3), and different ingredients act through different mechanisms in the human body. The combination of modern biomedical technology and TCM has shown a synergistic effect. Using omics and network pharmacology theory, researchers have gradually discovered the mechanisms of some Chinese medicines. Both *Lonicera japonica* (Thunb) and *Forsythia suspensa* (Thunb, Vahl) have significant synergistic benefits, conferring antipyretic and anti-free radical damage effects and improving immunity (Duan and Ma, 2009; Zhou et al., 2015). The mechanisms underlying the antipyretic effects of *Bupleurum chinense* (DC) and *Scutellaria baicalensis Georgi* are regulation of PGE2 and cAMP and inhibition of the synthesis or release of the endogenous pyrogens TNF-*α* and *β*-EP (Yang, 2012; Zhu et al., 2015; Yao et al., 2018). *Forsythia suspensa* (Thunb, Vahl) has been shown to have antipyretic effects (Wang and Zhao, 2010). One experiment confirmed that phillyrin, the active ingredient of *Forsythia suspensa* (Thunb, Vahl), can inhibit influenza A virus infection, most likely by downregulating a certain gene (Duan et al., 2012). Baicalin combined with phillyrin can downregulate the expression of the influenza A virus nucleoprotein gene transfected into HeLa cells, and it showed a synergistic effect within a certain concentration range (Huang et al., 2018). Studies have found that (R, S)-goitrin in *Isatis tinctoria L* inhibits influenza virus replication by increasing the production of IFN-β and interferon-induced transmembrane 3 (Luo et al., 2019). Modern pharmacological studies have found that *Isatis tinctoria L* has anti-inflammatory, anti-viral, antipyretic, and immunity enhancement effects, and is often used clinically to treat various infections, such as pharyngitis and tonsillitis (Yang, 2016). Studies have shown that the effect of *Platycodon grandiflorus* (Jacq.) A.DC in reducing phlegm and relieving cough is positively correlated with the content of platycodin (Xie et al., 2019). Platycodin capsules can reduce the frequency of cough and asthma, and increase the excretion of phenol red in the respiratory tract. The excretion of phenol red can indirectly reflect the amount of secretion in the respiratory tract. At this time, the amount of secretion in the respiratory tract increases, which is conducive to thinning the thick sputum attached to the respiratory tract mucosa, making it easier to fall off from the airway wall, which has the effect of reducing phlegm and relieving cough (Jin and Chen, 2015). Studies have shown that pulegone, the active ingredient of *Nepeta tenuifolia Benth*, has a good anti-inflammatory protective effect on Lipopolysaccharide poisoning model mice, which can inhibit the release of inflammatory factors and reduce inflammatory cell infiltration. The mechanism of its anti-inflammatory effect is related to the intervention and regulation of the activation of NLRP3 inflammasome (Wen, 2017). When influenza virus is combined with bacterial infection, combined prescription drugs can show the therapeutic advantage. The six CPMs included in the present study contain *Forsythia suspensa* (Thunb, Vahl). Among them, *Lianhua Qingwen* granules, *Xiaoer Resuqing* granules, and *Xiaoer Shuanghuanglian* mixture use *Lonicera japonica* (Thunb) and *Forsythia suspensa* (Thunb, Vahl) in combination. *Xiaoer Chiqiao Qingre* granules and *Xiaoer Resuqing* granules use *Bupleurum chinense* (DC) and *Scutellaria baicalensis Georgi* in combination. The drug composition of *Lianhua Qingwen* granules and *Xiaoer Resuqing* granules contains *Isatis tinctoria L*. *Nepeta tenuifolia Benth* is contained in the medicine composition of both *Xiaoer Chiqiao Qingre* granules and *Siji Kangbingdu* Mixture. By combining ancient theory and modern technology, researchers can possibly clarify the drug mechanisms of TCM, thereby guiding treatment with clinical prescription drugs.

In future clinical practice, combination medications may provide new ideas to treat children with influenza, promoting the use of CPMs and TCM for this purpose (Ru et al., 2017; Gao and Wang, 2018; Liu et al., 2019). They are especially effective at shortening the time to fever resolution and relieving the symptoms of influenza (Wang et al., 2010; Duan et al., 2011; Wang et al., 2011; He et al., 2019; Yoshino et al., 2019). This is consistent with the conclusions of the present study.

## Conclusion

In conclusion, the present meta-analysis demonstrated the efficacy and safety of CPMs combined with oseltamivir in the treatment of children with influenza. It provides evidence that combination therapy can be used to treat children with influenza. However, because most of the studies included in this analysis were of low quality and the analysis results showed high heterogeneity, there is no strong evidence to support this conclusion, which needs to be verified in future high-quality, well-designed, multi-center RCTs. At the same time, future studies should focus on improving objectivity in outcome measurements and methodological quality by adopting a rigorous experimental design. In future clinical research, the publication of RCTs should follow strict registration procedures, and standards should be unified as much as possible.

## Data Availability

The datasets presented in this study can be found in online repositories. The names of the repository/repositories and accession number(s) can be found in the article/Supplementary Material.
