# Supplementary material for: Efficacy and Safety of Chinese Patent Medicine Combined With Oseltamivir in Treatment of Children With Influenza: A meta-Analysis
Source: Front Pharmacol. 2021 Aug 6;12:682732. doi: 10.3389/fphar.2021.682732 (PMC8377812; doi:10.3389/fphar.2021.682732)
Supplement: Supplementary file 1 [file DataSheet1.zip › Supplementary materials/Figure 11.docx]

**Figure 11 Average time from symptom onset to randomization**

**distribution histogram**

Note: 1 to 18 represent 18 studies, blue represents the treatment group, and red represents the control group. The black line represents the standard deviation.
