# Supplementary material for: Efficacy and Safety of Chinese Patent Medicine Combined With Oseltamivir in Treatment of Children With Influenza: A meta-Analysis
Source: Front Pharmacol. 2021 Aug 6;12:682732. doi: 10.3389/fphar.2021.682732 (PMC8377812; doi:10.3389/fphar.2021.682732)
Supplement: Supplementary file 1 [file DataSheet1.zip › Supplementary materials/Table 2.docx]

**Table2 Mean difference (MD) and heterogeneity tests of outcomes for sensitivity analyses**

|  | | time to fever resolution | | | duration of cough | | | duration of nasal congestion | | | duration of sore throat | |
| --- | --- | --- | --- | --- | --- | --- | --- | --- | --- | --- | --- | --- |
| Excluded study | Pooled MD (95% CI) | | *Pheterogeneity;I^2^* | Pooled MD (95% CI) | | *Pheterogeneity;I^2^* | Pooled MD (95% CI) | | *Pheterogeneity;I^2^* | Pooled MD (95% CI) | | *Pheterogeneity;I^2^* |
| Liu 2020 | -0.64 (-0.88, -0.4) | | <0.00001; 99% | -0.85 (-1.08, -0.61) | | <0.00001; 94% | -0.94 (-1.27, -0.61) | | <0.00001; 97% | -0.98 (-1.39, -0.56) | | <0.00001; 98% |
| Long 2020 | -0.63 (-0.88, -0.39) | | <0.00001; 99% | -0.74 (-0.92, -0.57) | | <0.00001; 91% | -0.76 (-0.97, -0.56) | | <0.00001; 91% | -0.71 (-0.9, -0.52) | | <0.00001; 90% |
| Qian 2020 | -0.64 (-0.9, -0.38) | | <0.00001; 99% | -0.84 (-1.07, -0.61) | | <0.00001; 94% | -0.92 (-1.24, -0.6) | | <0.00001; 97% |  | |  |
| Song 2018 | -0.66 (-0.89, -0.43) | | <0.00001; 99% | -0.83 (-1.04, -0.62) | | <0.00001; 94% |  | |  | -0.98 (-1.35, -0.6) | | <0.00001; 98% |
| Su 2019 | -0.59 (-0.81, -0.36) | | <0.00001; 99% | -0.77 (-0.96, -0.57) | | <0.00001; 93% | -0.88 (-1.17, -0.59) | | <0.00001; 97% |  | |  |
| Wu 2020 | -0.63 (-0.87, -0.4) | | <0.00001; 99% |  | |  | -0.79 (-1.07, -0.52) | | <0.00001; 96% | -0.8 (-1.15, -0.46) | | <0.00001; 98% |
| Yin 2019 | -0.63 (-0.86, -0.39) | | <0.00001; 99% | -0.77 (-0.97, -0.57) | | <0.00001; 94% |  | |  | -0.91 (-1.27, -0.56) | | <0.00001; 98% |
| Zhang 2018 | -0.65 (-0.88, -0.42) | | <0.00001; 99% | -0.85 (-1.07, -0.64) | | <0.00001; 94% | -0.94 (-1.24, -0.64) | | <0.00001; 97% | -0.97 (-1.33, -0.6) | | <0.00001; 98% |
| Zhangy 2018 | -0.66 (-0.89, -0.42) | | <0.00001; 99% |  | |  |  | |  |  | |  |
| Zhao 2019 | -0.64 (-0.89, -0.39) | | <0.00001; 99% | -0.78 (-0.98, -0.57) | | <0.00001; 94% | -0.89 (-1.17, -0.6) | | <0.00001; 97% |  | |  |
| Zhou 2019 | -0.61 (-0.84, -0.38) | | <0.00001; 99% |  | |  |  | |  |  | |  |
| Chen 2019 | -0.65 (-0.88, -0.42) | | <0.00001; 99% |  | |  |  | |  |  | |  |
| Li 2019 | -0.55 (-0.67, -0.43) | | <0.00001; 95% |  | |  |  | |  |  | |  |
| Liu 2020 | -0.68 (-0.9, -0.45) | | <0.00001; 99% | -0.82 (-1.03, -0.62) | | <0.00001; 94% |  | |  | -0.9 (-1.27, -0.54) | | <0.00001; 98% |
| Zhu 2019 | -0.64 (-0.87, -0.41) | | <0.00001; 99% | -0.83 (-1.04, -0.61) | | <0.00001; 94% |  | |  | -0.98 (-1.37, -0.6) | | <0.00001; 98% |
| Fang 2018 | -0.66 (-0.89, -0.42) | | <0.00001; 99% |  | |  |  | |  |  | |  |
| Gao 2020 | -0.64 (-0.87, -0.41) | | <0.00001; 99% | -0.82 (-1.03, -0.61) | | <0.00001; 94% |  | |  |  | |  |
| Kuang 2020 | -0.67 (-0.89, -0.44) | | <0.00001; 99% | -0.82 (-1.03, -0.6) | | <0.00001; 94% | -0.88 (-1.15, -0.61) | | <0.00001; 96% |  | |  |
| Du 2020 | -0.66 (-0.89, -0.43) | | <0.00001; 99% | -0.86 (-1.07, -0.66) | | <0.00001; 94% | -0.9 (-1.19, -0.61) | | <0.00001; 97% | -0.97 (-1.34, -0.61) | | <0.00001; 98% |
| Liu 2018 |  | |  | -0.87 (-1.06, -0.67) | | <0.00001; 92% | -0.95 (-1.23, -0.68) | | <0.00001; 95% | -0.98 (-1.38, -0.58) | | <0.00001; 98% |
| Yan 2020 |  | |  |  | |  | -0.8 (-1.07, -0.53) | | <0.00001; 96% | -0.83 (-1.18, -0.48) | | <0.00001; 98% |
