# Supplementary material for: Efficacy and Safety of Chinese Patent Medicine Combined With Oseltamivir in Treatment of Children With Influenza: A meta-Analysis
Source: Front Pharmacol. 2021 Aug 6;12:682732. doi: 10.3389/fphar.2021.682732 (PMC8377812; doi:10.3389/fphar.2021.682732)
Supplement: Supplementary file 1 [file DataSheet1.zip › Supplementary materials/Table3.docx]

**Table3 The general characters of CPMs.**

| **CPMs** | **Study** | **Formulation, Source, Species, concentration** | **Quality control reported? (Y/N)** | **Chemical**  **analysis**  **reported? (Y/N)** | **phytochemical constituents used for HPLC analysis** |
| --- | --- | --- | --- | --- | --- |
| Xiaoer Chiqiao Qingre Granules | Song 2018, Zhang  2018, Zhangy 2018, Su 2019, Yin 2019, Zhou 2019, Zhao 2019, Long 2020, Liu 2020, Qian 2020, Wu 2020 | **[Jiang-su Jichuan pharmaceutical co., Ltd]**. (1) *[Forsythia suspensa](https://mpns.science.kew.org/mpns-portal/plantDetail?plantId=369441&query=%E8%BF%9E%E7%BF%98&filter=&fuzzy=false&nameType=all&dbs=wcs)* [(Thunb.) Vahl](https://mpns.science.kew.org/mpns-portal/plantDetail?plantId=369441&query=%E8%BF%9E%E7%BF%98&filter=&fuzzy=false&nameType=all&dbs=wcs) 444g (2) *Glycine max* (L.) Merr. 333g (3) *[Mentha canadensis L.](https://mpns.science.kew.org/mpns-portal/plantDetail?plantId=124520&query=%E8%96%84%E8%8D%B7&filter=&fuzzy=false&nameType=all&dbs=wcs)* 222g (4) *[Nepeta tenuifolia Benth.](https://mpns.science.kew.org/mpns-portal/plantDetail?plantId=135025&query=%E8%8D%86%E8%8A%A5&filter=&fuzzy=false&nameType=all&dbs=wcs)* 222g (5) *[Gardenia jasminoides J.Ellis](https://mpns.science.kew.org/mpns-portal/plantDetail?plantId=88270&query=%E6%A0%80%E5%AD%90&filter=&fuzzy=false&nameType=all&dbs=wcs)* 189g (6) *Rheum officinale Baill*. 189g (7) *[Artemisia annua L](https://mpns.science.kew.org/mpns-portal/plantDetail?plantId=901442&query=%E9%9D%92%E8%92%BF&filter=&fuzzy=false&nameType=all&dbs=wcsCmp)*[.](https://mpns.science.kew.org/mpns-portal/plantDetail?plantId=901442&query=%E9%9D%92%E8%92%BF&filter=&fuzzy=false&nameType=all&dbs=wcsCmp) 333g (8) *[Paeonia anomala subsp](https://mpns.science.kew.org/mpns-portal/plantDetail?plantId=518977&query=%E8%B5%A4%E8%8A%8D&filter=&fuzzy=false&nameType=all&dbs=wcs)*[.](https://mpns.science.kew.org/mpns-portal/plantDetail?plantId=518977&query=%E8%B5%A4%E8%8A%8D&filter=&fuzzy=false&nameType=all&dbs=wcs) *[veitchii](https://mpns.science.kew.org/mpns-portal/plantDetail?plantId=518977&query=%E8%B5%A4%E8%8A%8D&filter=&fuzzy=false&nameType=all&dbs=wcs)* [(Lynch) D.Y.Hong & K.Y.Pan](https://mpns.science.kew.org/mpns-portal/plantDetail?plantId=518977&query=%E8%B5%A4%E8%8A%8D&filter=&fuzzy=false&nameType=all&dbs=wcs) 222g (9) *Areca catechu L*. 167g (10) *[Magnolia officinalis](https://mpns.science.kew.org/mpns-portal/plantDetail?plantId=117741&query=%E5%8E%9A%E6%9C%B4&filter=&fuzzy=false&nameType=all&dbs=wcs)* [Rehder & E.H.Wilson](https://mpns.science.kew.org/mpns-portal/plantDetail?plantId=117741&query=%E5%8E%9A%E6%9C%B4&filter=&fuzzy=false&nameType=all&dbs=wcs) 333g (11) *Scutellaria baicalensis Georgi* 333g (12) *Pinellia ternata* (Thunb.) Makino 333g (13) *Bupleurum* chinense DC. 222g (14) *Glycyrrhiza uralensis Fisch.* ex DC. 189g | Y-Prepared  according to  Chinese  pharmacopoeia | Y - HPLC | phillyrin, daidzein and genistein, pulegone, gardenoside, anthraquinone, paeony glycoside, arecoline, magnolol and honokiol, baicalin, saikosaponin a and saikosaponin d, liquiritin and glycyrrhizic acid |
| Kanggan Granules | Chen 2019, Li 2019, Yan 2020 | **[Sichuan Good Doctor Panxi Pharmaceutical Co. Ltd (Chen 2019, Li 2019) or Sichuan Tongrentai Pharmaceutical Co. Ltd (Yan 2020)].** (1) *Lonicera japonica* Thunb. 700g (2) *[Paeonia anomala subsp. veitchii](https://mpns.science.kew.org/mpns-portal/plantDetail?plantId=518977&query=%E8%B5%A4%E8%8A%8D&filter=&fuzzy=false&nameType=all&dbs=wcs)* [(Lynch) D.Y.Hong & K.Y.Pan](https://mpns.science.kew.org/mpns-portal/plantDetail?plantId=518977&query=%E8%B5%A4%E8%8A%8D&filter=&fuzzy=false&nameType=all&dbs=wcs) 700g (3) *Cyrtomium fortunei J.Sm*. 233g | Y-Prepared  according to  Chinese  pharmacopoeia | Y - HPLC | galuteolin, paeony glycoside |
| Lianhua Qingwen Granules | Zhu 2019, Liu 2020 | **[Shijiazhuang Yiling Pharmaceutical Co., Ltd (Zhu 2019) or Beijing Yiling Pharmaceutical Co., Ltd (Liu 2020)]**. (1) *[Forsythia suspensa](https://mpns.science.kew.org/mpns-portal/plantDetail?plantId=369441&query=%E8%BF%9E%E7%BF%98&filter=&fuzzy=false&nameType=all&dbs=wcs)* [(Thunb.) Vahl](https://mpns.science.kew.org/mpns-portal/plantDetail?plantId=369441&query=%E8%BF%9E%E7%BF%98&filter=&fuzzy=false&nameType=all&dbs=wcs) 170g (2) *Lonicera japonica* Thunb. 170g (3) *Ephedra sinica Stapf* 57g (4) *Prunus armeniaca L*. 57g (5) *shi gao* (Gypsum) 170g (6) *Isatis tinctoria L*. 170g (7) *Cyrtomium fortunei J.Sm*. 170g (8) *Houttuynia cordata* Thunb. 170g (9) *Pogostemon cablin* (Blanco) Benth. 57g (10) *Rheum officinale Baill*. 34g (11) *[Rhodiola](https://mpns.science.kew.org/mpns-portal/plantDetail?plantId=416737&query=%E7%BA%A2%E6%99%AF%E5%A4%A9&filter=&fuzzy=false&nameType=all&dbs=wcsCmp)**[crenulata](https://mpns.science.kew.org/mpns-portal/plantDetail?plantId=416737&query=%E7%BA%A2%E6%99%AF%E5%A4%A9&filter=&fuzzy=false&nameType=all&dbs=wcsCmp)* [(Hook.f. & Thomson) H.Ohba](https://mpns.science.kew.org/mpns-portal/plantDetail?plantId=416737&query=%E7%BA%A2%E6%99%AF%E5%A4%A9&filter=&fuzzy=false&nameType=all&dbs=wcsCmp) 57g (12) *[Mentha canadensis L](https://mpns.science.kew.org/mpns-portal/plantDetail?plantId=124520&query=%E8%96%84%E8%8D%B7&filter=&fuzzy=false&nameType=all&dbs=wcs)*[.](https://mpns.science.kew.org/mpns-portal/plantDetail?plantId=124520&query=%E8%96%84%E8%8D%B7&filter=&fuzzy=false&nameType=all&dbs=wcs) 5g (13) *Glycyrrhiza uralensis Fisch*. ex DC. 57g | Y-Prepared  according to  Chinese  pharmacopoeia | Y - HPLC | phillyrin, galuteolin, ephedrine and pseudoephedrine, amygdalin, (R, S) - goitrin, anthraquinone, rhodioloside, liquiritin and glycyrrhizic acid |
| Xiaoer Resuqing Granules (oral liquid) | Fang 2018, Liu 2018 | **[Jiangxi Beiken Pharmaceutical Co. Ltd (Fang 2018) or Jilin Yizheng Pharmaceutical Group Co. Ltd (Liu 2018)]**. Xiaoer Resuqing Granules: (1) *Bupleurum chinense DC*. 1250g (2) *Scutellaria baicalensis Georgi* 625g (3) *Isatis tinctoria L*. 1250g (4) *Pueraria montana var. Lobata* (Willd.) Maesen & S.M.Almeida ex Sanjappa & Predeep 625g (5) *Lonicera japonica* Thunb. 687.5g (6) *shui niu jiao* (Bubali cornu) 312.5g (7) *[Forsythia suspensa](https://mpns.science.kew.org/mpns-portal/plantDetail?plantId=369441&query=%E8%BF%9E%E7%BF%98&filter=&fuzzy=false&nameType=all&dbs=wcs)* [(Thunb.) Vahl](https://mpns.science.kew.org/mpns-portal/plantDetail?plantId=369441&query=%E8%BF%9E%E7%BF%98&filter=&fuzzy=false&nameType=all&dbs=wcs) 750g (8) *Rheum officinale Baill*. 312.5g. Xiaoer Resuqing oral liquid: (1) *Bupleurum chinense DC*. 250g (2) *Scutellaria baicalensis Georgi* 125g (3) *Isatis tinctoria L*. 250g (4) *Pueraria montana* var. Lobata (Willd.) Maesen & S.M.Almeida ex Sanjappa & Predeep 125g (5) *Lonicera japonica* Thunb. 137.5g (6) *shui niu jiao* (Bubali cornu) 62.5g (7) *[Forsythia suspensa](https://mpns.science.kew.org/mpns-portal/plantDetail?plantId=369441&query=%E8%BF%9E%E7%BF%98&filter=&fuzzy=false&nameType=all&dbs=wcs)* [(Thunb.) Vahl](https://mpns.science.kew.org/mpns-portal/plantDetail?plantId=369441&query=%E8%BF%9E%E7%BF%98&filter=&fuzzy=false&nameType=all&dbs=wcs) 150g (8) *Rheum officinale Baill*. 62.5g | Y-Prepared  according to  Chinese  pharmacopoeia | Y - HPLC | saikosaponin a and saikosaponin d, baicalin, (R, S) - goitrin, puerarin, galuteolin, phillyrin, anthraquinone |
| Xiaoer Shuanghuanglian Mixture | Gao 2020 | **[Henan Tailong Pharmaceutical Co. Ltd]**. (1) *Lonicera japonica* Thunb. 375g (2) *Scutellaria baicalensis Georgi* 375g (3) *[Forsythia suspensa](https://mpns.science.kew.org/mpns-portal/plantDetail?plantId=369441&query=%E8%BF%9E%E7%BF%98&filter=&fuzzy=false&nameType=all&dbs=wcs)* [(Thunb.) Vahl](https://mpns.science.kew.org/mpns-portal/plantDetail?plantId=369441&query=%E8%BF%9E%E7%BF%98&filter=&fuzzy=false&nameType=all&dbs=wcs) 750g | Y-Prepared  according to  Chinese  pharmacopoeia | Y - HPLC | galuteolin, baicalin, phillyrin |
| Siji Kangbingdu Mixture | Kuang 2020 | **[Shaanxi Haitian Pharmaceutical Co. Ltd]**. (1) *Houttuynia cordata* Thunb. (2) *Platycodon grandiflorus* (Jacq.) A.DC. (3) *Morus alba L*. (4) *[Forsythia suspensa](https://mpns.science.kew.org/mpns-portal/plantDetail?plantId=369441&query=%E8%BF%9E%E7%BF%98&filter=&fuzzy=false&nameType=all&dbs=wcs)* [(Thunb.) Vahl](https://mpns.science.kew.org/mpns-portal/plantDetail?plantId=369441&query=%E8%BF%9E%E7%BF%98&filter=&fuzzy=false&nameType=all&dbs=wcs) (5) *Nepeta tenuifolia Benth*. (6) *[Mentha canadensis L](https://mpns.science.kew.org/mpns-portal/plantDetail?plantId=124520&query=%E8%96%84%E8%8D%B7&filter=&fuzzy=false&nameType=all&dbs=wcs)*[.](https://mpns.science.kew.org/mpns-portal/plantDetail?plantId=124520&query=%E8%96%84%E8%8D%B7&filter=&fuzzy=false&nameType=all&dbs=wcs) (7) *Perilla frutescens* (L.) *Britton* (8) *Prunus armeniaca L*. (9) *Phragmites australis* (Cav.) Trin. ex Steud. (10) *Chrysanthemum × morifolium* (Ramat.) Hemsl. (11) *Glycyrrhiza uralensis Fisch.* ex DC. Formulation dosage not found. | NA | Y - HPLC | platycodin, rutin, phillyrin, pulegone, amygdalin, chlorogenic acid and galuteolin, liquiritin and glycyrrhizic acid |
| Xiaoer Niuhuang Qingxin Powder | Du 2020 | **[Shandong Guangyutang Traditional Chinese Medicine Co. Ltd]**. (1) *Gastrodia elata Blume* (2) *[Arisaema erubescens](https://mpns.science.kew.org/mpns-portal/plantDetail?plantId=15355&query=%E5%8D%97%E6%98%9F&filter=&fuzzy=false&nameType=all&dbs=wcs)* [(Wall.) Schott](https://mpns.science.kew.org/mpns-portal/plantDetail?plantId=15355&query=%E5%8D%97%E6%98%9F&filter=&fuzzy=false&nameType=all&dbs=wcs) (3) *Coptis chinensis Franch*. (4) *[Paeonia anomala subsp](https://mpns.science.kew.org/mpns-portal/plantDetail?plantId=518977&query=%E8%B5%A4%E8%8A%8D&filter=&fuzzy=false&nameType=all&dbs=wcs)*[. veitchii (Lynch) D.Y.Hong & K.Y.Pan](https://mpns.science.kew.org/mpns-portal/plantDetail?plantId=518977&query=%E8%B5%A4%E8%8A%8D&filter=&fuzzy=false&nameType=all&dbs=wcs) (5) *Rheum officinale Baill.* (6) *quan xie* (Scorpio) (7) *shui niu jiao* (Bubali cornu) (8) *jiang can* (Bombyx batryticatus) (9) *niu huang* (Bovis calculus) (10) *hu po* (Succinum) (11) *xiong huang* (Realgar) (12) *Dryobalanops aromatica C.F.Gaertn*. (13) *zhu sha* (Cinnabaris) (14) *jin meng shi* (Micae lapis aureus). Formulation dosage not found. | NA | Y - HPLC | gastrodine and p-hydroxybenzyl alcohol, berberine hydrochloride, paeony glycoside, anthraquinone, bilirubin |
